# (*E*)-1-(4,4′′-Difluoro-5′-meth­oxy-1,1′:3′,1′′-terphenyl-4′-yl)-3-(4-nitro­phen­yl)prop-2-en-1-one

**DOI:** 10.1107/S1600536811045806

**Published:** 2011-11-05

**Authors:** Richard Betz, Thomas Gerber, Eric Hosten, S. Samshuddin, Badiadka Narayana, Hemmige S. Yathirajan

**Affiliations:** a Nelson Mandela Metropolitan University, Summerstrand Campus, Department of Chemistry, University Way, Summerstrand, PO Box 77000, Port Elizabeth 6031, South Africa; b Mangalore University, Department of Studies in Chemistry, Mangalagangotri 574 199, India; c University of Mysore, Department of Studies in Chemistry, Manasagangotri, Mysore 570 006, India

## Abstract

In the title compound, C_28_H_19_F_2_NO_4_, a polysubstituted terphenyl derivative bearing a Michael system, the C=C double bond has an *E* configuration. Two C—H⋯F contacts connect mol­ecules into inversion dimers. In addition, a C–H⋯π as well as a C–F⋯π contact can be identified. The shortest centroid–centroid distance between two aromatic rings is 3.9535 (8) Å, between one of the *para*-fluoro­benzene rings and its symmetry-generated equivalent.

## Related literature

For the pharmacological importance of terphenyls, see: Liu (2006 ▶) and of chalcones, see: Dhar (1981 ▶); Dimmock *et al.* (1999 ▶); Satyanarayana *et al.* (2004 ▶). For our work on the synthesis of different chalcone derivatives, see: Samshuddin *et al.* (2011*a* ▶,*b* ▶); Fun *et al.* (2010*a* ▶,*b* ▶); Jasinski *et al.* (2010*a* ▶,*b* ▶); Baktır *et al.* (2011*a* ▶,*b* ▶). For graph-set analysis of hydrogen bonds, see: Etter *et al.* (1990 ▶); Bernstein *et al.* (1995 ▶).
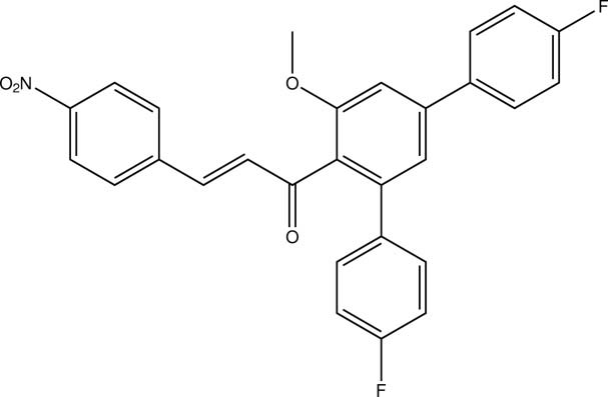


## Experimental

### 

#### Crystal data

C_28_H_19_F_2_NO_4_
*M* *_r_* = 471.44
Monoclinic, 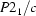

*a* = 23.3751 (7) Å
*b* = 6.9098 (2) Å
*c* = 13.7879 (5) Å
β = 99.243 (2)°
*V* = 2198.07 (12) Å^3^
*Z* = 4
Mo *K*α radiation
μ = 0.11 mm^−1^
*T* = 200 K
0.58 × 0.44 × 0.17 mm

#### Data collection

Bruker APEXII CCD diffractometer
37111 measured reflections
5462 independent reflections
4899 reflections with *I* > 2σ(*I*)
*R*_int_ = 0.026

#### Refinement

*R*[*F* ^2^ > 2σ(*F* ^2^)] = 0.045
*wR*(*F* ^2^) = 0.114
*S* = 1.05
5462 reflections
317 parameters
H-atom parameters constrained
Δρ_max_ = 0.32 e Å^−3^
Δρ_min_ = −0.24 e Å^−3^

### 

Data collection: *APEX2* (Bruker, 2010 ▶); cell refinement: *SAINT* (Bruker, 2010 ▶); data reduction: *SAINT*; program(s) used to solve structure: *SHELXS97* (Sheldrick, 2008 ▶); program(s) used to refine structure: *SHELXL97* (Sheldrick, 2008 ▶); molecular graphics: *ORTEP-3* (Farrugia, 1997 ▶) and *Mercury* (Macrae *et al.*, 2008 ▶); software used to prepare material for publication: *SHELXL97* and *PLATON* (Spek, 2009 ▶).

## Supplementary Material

Crystal structure: contains datablock(s) I, global. DOI: 10.1107/S1600536811045806/fj2465sup1.cif

Supplementary material file. DOI: 10.1107/S1600536811045806/fj2465Isup2.cdx

Structure factors: contains datablock(s) I. DOI: 10.1107/S1600536811045806/fj2465Isup3.hkl

Supplementary material file. DOI: 10.1107/S1600536811045806/fj2465Isup4.cml

Additional supplementary materials:  crystallographic information; 3D view; checkCIF report

## Figures and Tables

**Table 1:** Hydrogen-bond geometry (Å, °) *Cg*1 is the centroid of the C11–C16 ring.

*D*—H⋯*A*	*D*—H	H⋯*A*	*D*⋯*A*	*D*—H⋯*A*
C25—H25⋯F1^i^	0.95	2.54	3.2165 (17)	129
C33—H33⋯*Cg*1^ii^	0.95	2.91	3.4748 (15)	119
C24—F1⋯*Cg*1^iii^	1.36 (1)	3.95 (1)	4.8373 (15)	123 (1)

Symmetry codes: (i) 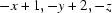; (ii) 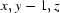; (iii) 
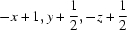
.

**Fig. 1.**
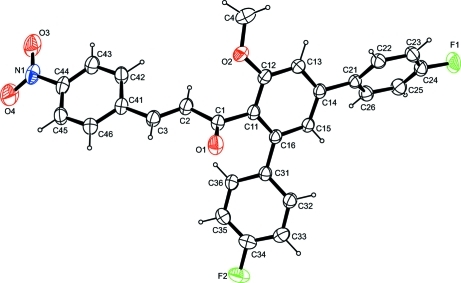
The molecular structure of the title compound, with atom labels and anisotropic displacement ellipsoids (drawn at 50% probability level).

**Fig. 2.**
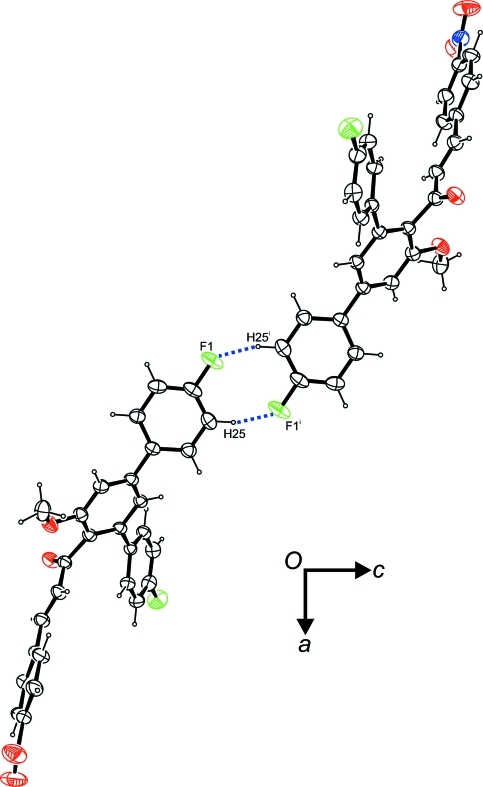
Intermolecular contacts, viewed along [0 1 0]. Symmetry operator: i -x + 1, -y + 2, -z.

**Fig. 3.**
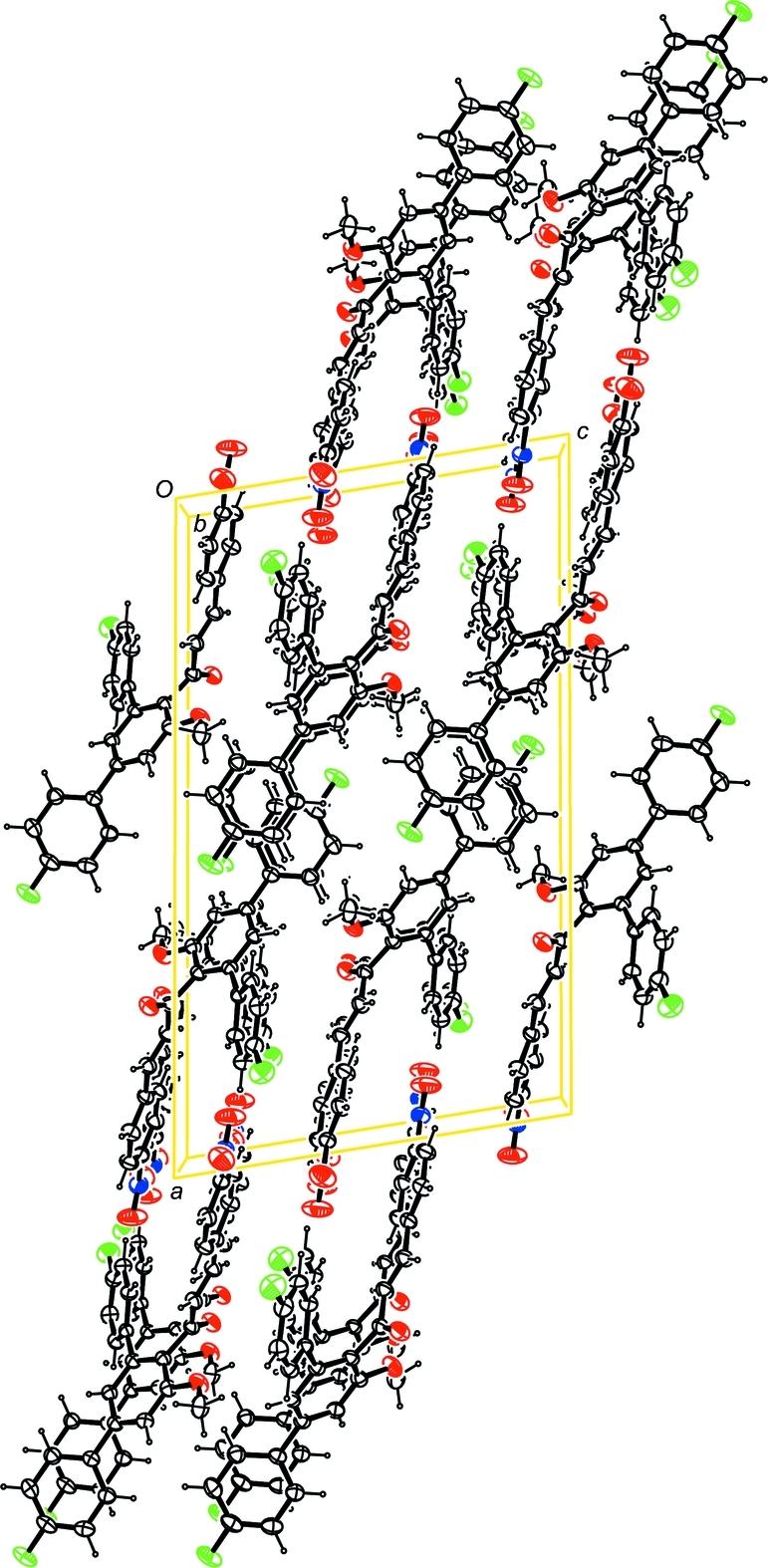
Molecular packing of the title compound, viewed along [0 1 0] (anisotropic displacement ellipsoids drawn at 50% probability level).

## supplementary crystallographic information

### Comment

Chalcones constitute an important family of substances belonging to flavonoids, a large group of natural and synthetic products with interesting physicochemical properties, biological activity and structural characteristics. They have been reported to possess many interesting pharmacological activities (Dhar, 1981) including anti-inflammatory, antimicrobial, antifungal, antioxidant, cytotoxic, antitumor and anticancer activities (Dimmock *et al.*, 1999; Satyanarayana *et al.*, 2004). In recent years, it has been reported that some terphenyls exhibit considerable biological activities (*e.g.* being potent anticoagulants, immunosuppressants, antithrombotics, neuroprotectives, specific 5-lipoxygenase inhibitors) and showing cytotoxic activities (Liu, 2006). In view of the pharmacological importance of terphenyls and chalcones, and in continuation of our work on synthesis of various derivatives of 4,4'-difluoro chalcone (Samshuddin *et al.*, 2011*a*/b, Fun *et al.*, 2010*a*/b, Jasinski *et al.*, 2010*a*/b, Baktır *et al.*, 2011*a*/b), the molecular and crystal structure of the title compound is reported.

The C=C double of the Michael system is (*E*)-configured. The least-squares planes defined by the carbon atoms of the *para*-fluoro phenyl rings of the terphenyl moiety and its central phenyl ring enclose angles of 40.43 (6)° and 43.99 (6)°, respectively. The plane defined by the atoms of the nitro group is tilted by 13.56 (19)° with respect to the plane of the aromatic system it is bonded to (Fig. 1).

In the crystal, C–H···F contacts whose range falls by more than 0.1 Å below the sum of van-der-Waals radii of the corresponding atoms are observed. These are supported by one of the hydrogen atoms in *ortho* position to a fluorine atom whose symmetry-generated equivalent acts as acceptor for this type of contact. In total, the molecules are connected to centrosymmetric dimers (Fig. 2). In terms of graph-set analysis (Etter *et al.*, 1990; Bernstein *et al.*, 1995), the descriptor for the C–H···F contacts is *R*^2^_2_(8) on the unitary level. In addition, a C–H···π as well as a C–F···π contact can be identified. The shortest intercentroid distance between two aromatic systems is apparent between two of the *para*-fluoro phenyl moieties that are also part of the C–H···F contacts and was measured at 3.9535 (8) Å. Details about metrical parameters of the intermolecular contacts and their symmetry can be found in Table 1.

The packing of the title compound in the crystal is shown in Figure 3.

### Experimental

To a mixture of 1-(4,4''-difluoro-5'-methoxy-1,1':3',1''-terphenyl-4'-yl) ethanone (3.38 g, 0.01 mol) and 4-nitrobenzaldehyde (1.51 g, 0.01 mol) in 40 ml of ethanol, 10 ml of 10% sodium hydroxide solution was added and stirred at 5–10 °C for 3 h. The precipitate formed was collected by filtration and purified by recrystallization from ethanol (yield: 80%). Single crystals suitable for the X-ray diffraction study were grown from DMF–ethanol (*v*:*v* 1:1) by slow evaporation at room temperature.

### Refinement

Carbon-bound H atoms were placed in calculated positions (C—H 0.95 Å for aromatic and vinylic carbon atoms, C—H 0.99 Å for methylene groups) and were included in the refinement in the riding model approximation, with *U*(H) set to 1.2*U*_eq_(C). The H atoms of the methyl groups were allowed to rotate with a fixed angle around the C—C bond to best fit the experimental electron density (HFIX 137 in the *SHELX* program suite (Sheldrick, 2008)), with *U*(H) set to 1.5*U*_eq_(C).

### Crystal data

**Table:**

C_28_H_19_F_2_NO_4_	*F*(000) = 976
*M**_r_* = 471.44	*D*_x_ = 1.425 Mg m^−^^3^
Monoclinic, *P*2_1_/*c*	Melting point: 489 K
Hall symbol: -P 2ybc	Mo *K*α radiation, λ = 0.71073 Å
*a* = 23.3751 (7) Å	Cell parameters from 9792 reflections
*b* = 6.9098 (2) Å	θ = 2.7–28.4°
*c* = 13.7879 (5) Å	µ = 0.11 mm^−^^1^
β = 99.243 (2)°	*T* = 200 K
*V* = 2198.07 (12) Å^3^	Block, yellow
*Z* = 4	0.58 × 0.44 × 0.17 mm

### Data collection

**Table:**

Bruker APEXII CCD diffractometer	4899 reflections with *I* > 2σ(*I*)
Radiation source: fine-focus sealed tube	*R*_int_ = 0.026
graphite	θ_max_ = 28.4°, θ_min_ = 1.8°
φ and ω scans	*h* = −31→31
37111 measured reflections	*k* = −9→9
5462 independent reflections	*l* = −18→18

### Refinement

**Table:**

Refinement on *F*^2^	Primary atom site location: structure-invariant direct methods
Least-squares matrix: full	Secondary atom site location: difference Fourier map
*R*[*F*^2^ > 2σ(*F*^2^)] = 0.045	Hydrogen site location: inferred from neighbouring sites
*wR*(*F*^2^) = 0.114	H-atom parameters constrained
*S* = 1.05	*w* = 1/[σ^2^(*F*_o_^2^) + (0.0442*P*)^2^ + 1.0552*P*] where *P* = (*F*_o_^2^ + 2*F*_c_^2^)/3
5462 reflections	(Δ/σ)_max_ < 0.001
317 parameters	Δρ_max_ = 0.32 e Å^−^^3^
0 restraints	Δρ_min_ = −0.24 e Å^−^^3^

### Fractional atomic coordinates and isotropic or equivalent isotropic displacement parameters (Å^2^)

**Table:**

	*x*	*y*	*z*	*U*_iso_*/*U*_eq_	
F1	0.54918 (4)	0.89243 (16)	0.09405 (8)	0.0577 (3)	
F2	0.14304 (5)	0.02099 (15)	0.26896 (8)	0.0585 (3)	
O1	0.25621 (5)	0.57681 (15)	0.56774 (8)	0.0428 (2)	
O2	0.32555 (5)	1.02608 (15)	0.54905 (8)	0.0426 (2)	
O3	−0.01724 (6)	1.5042 (2)	0.61968 (11)	0.0651 (4)	
O4	−0.06990 (5)	1.2537 (2)	0.63545 (13)	0.0765 (5)	
N1	−0.02455 (6)	1.3295 (2)	0.62244 (10)	0.0480 (3)	
C1	0.25169 (5)	0.72612 (19)	0.52066 (9)	0.0310 (3)	
C2	0.20726 (6)	0.8734 (2)	0.53077 (10)	0.0346 (3)	
H2	0.2095	0.9969	0.5013	0.042*	
C3	0.16442 (6)	0.8363 (2)	0.58026 (10)	0.0348 (3)	
H3	0.1650	0.7142	0.6121	0.042*	
C4	0.35123 (8)	1.2132 (2)	0.55848 (11)	0.0460 (3)	
H4A	0.3935	1.2006	0.5682	0.069*	
H4B	0.3397	1.2798	0.6151	0.069*	
H4C	0.3382	1.2882	0.4987	0.069*	
C11	0.29155 (5)	0.76687 (18)	0.44743 (9)	0.0291 (2)	
C12	0.33024 (6)	0.92135 (18)	0.46656 (9)	0.0314 (3)	
C13	0.37199 (5)	0.95644 (19)	0.40751 (9)	0.0314 (3)	
H13	0.3990	1.0591	0.4228	0.038*	
C14	0.37401 (5)	0.83985 (18)	0.32563 (9)	0.0287 (2)	
C15	0.33356 (5)	0.69187 (18)	0.30343 (9)	0.0284 (2)	
H15	0.3338	0.6170	0.2457	0.034*	
C16	0.29257 (5)	0.65097 (17)	0.36424 (9)	0.0274 (2)	
C21	0.41994 (5)	0.86685 (17)	0.26396 (9)	0.0292 (2)	
C22	0.47750 (6)	0.89612 (19)	0.30685 (10)	0.0338 (3)	
H22	0.4870	0.9087	0.3762	0.041*	
C23	0.52104 (6)	0.9071 (2)	0.24956 (11)	0.0386 (3)	
H23	0.5602	0.9277	0.2787	0.046*	
C24	0.50613 (6)	0.8877 (2)	0.14999 (11)	0.0394 (3)	
C25	0.45022 (7)	0.8625 (2)	0.10411 (11)	0.0410 (3)	
H25	0.4413	0.8524	0.0346	0.049*	
C26	0.40697 (6)	0.8522 (2)	0.16222 (10)	0.0356 (3)	
H26	0.3678	0.8348	0.1319	0.043*	
C31	0.25194 (5)	0.48643 (18)	0.33831 (9)	0.0278 (2)	
C32	0.27220 (6)	0.31378 (19)	0.30383 (10)	0.0331 (3)	
H32	0.3119	0.3038	0.2968	0.040*	
C33	0.23598 (6)	0.1566 (2)	0.27957 (10)	0.0384 (3)	
H33	0.2501	0.0399	0.2556	0.046*	
C34	0.17884 (6)	0.1751 (2)	0.29133 (11)	0.0390 (3)	
C35	0.15653 (6)	0.3426 (2)	0.32347 (10)	0.0381 (3)	
H35	0.1168	0.3510	0.3300	0.046*	
C36	0.19314 (5)	0.4991 (2)	0.34611 (10)	0.0329 (3)	
H36	0.1781	0.6168	0.3672	0.039*	
C41	0.11626 (6)	0.9660 (2)	0.59044 (9)	0.0334 (3)	
C42	0.11732 (6)	1.1622 (2)	0.56648 (11)	0.0388 (3)	
H42	0.1500	1.2140	0.5423	0.047*	
C43	0.07152 (6)	1.2819 (2)	0.57750 (11)	0.0407 (3)	
H43	0.0725	1.4158	0.5623	0.049*	
C44	0.02418 (6)	1.2015 (2)	0.61122 (10)	0.0378 (3)	
C45	0.02118 (6)	1.0089 (2)	0.63473 (11)	0.0414 (3)	
H45	−0.0122	0.9574	0.6568	0.050*	
C46	0.06796 (6)	0.8922 (2)	0.62547 (11)	0.0395 (3)	
H46	0.0672	0.7595	0.6433	0.047*	

### Atomic displacement parameters (Å^2^)

**Table:**

	*U*^11^	*U*^22^	*U*^33^	*U*^12^	*U*^13^	*U*^23^
F1	0.0536 (6)	0.0593 (6)	0.0704 (6)	0.0013 (5)	0.0407 (5)	0.0106 (5)
F2	0.0576 (6)	0.0439 (5)	0.0721 (7)	−0.0193 (4)	0.0047 (5)	−0.0043 (5)
O1	0.0448 (5)	0.0411 (5)	0.0462 (6)	0.0121 (4)	0.0186 (4)	0.0126 (4)
O2	0.0513 (6)	0.0372 (5)	0.0438 (5)	−0.0048 (4)	0.0208 (5)	−0.0128 (4)
O3	0.0632 (8)	0.0562 (8)	0.0811 (9)	0.0269 (6)	0.0276 (7)	0.0082 (7)
O4	0.0345 (6)	0.0864 (11)	0.1117 (12)	0.0079 (6)	0.0207 (7)	−0.0264 (9)
N1	0.0370 (6)	0.0639 (9)	0.0438 (7)	0.0154 (6)	0.0084 (5)	−0.0087 (6)
C1	0.0300 (6)	0.0330 (6)	0.0312 (6)	0.0043 (5)	0.0084 (5)	−0.0003 (5)
C2	0.0362 (6)	0.0341 (6)	0.0355 (6)	0.0077 (5)	0.0118 (5)	0.0020 (5)
C3	0.0340 (6)	0.0346 (7)	0.0373 (6)	0.0057 (5)	0.0102 (5)	0.0005 (5)
C4	0.0619 (9)	0.0356 (7)	0.0392 (7)	0.0010 (7)	0.0041 (7)	−0.0078 (6)
C11	0.0281 (5)	0.0288 (6)	0.0316 (6)	0.0060 (5)	0.0085 (4)	0.0021 (5)
C12	0.0337 (6)	0.0283 (6)	0.0331 (6)	0.0051 (5)	0.0082 (5)	−0.0022 (5)
C13	0.0303 (6)	0.0278 (6)	0.0368 (6)	0.0015 (5)	0.0071 (5)	−0.0007 (5)
C14	0.0266 (5)	0.0281 (6)	0.0322 (6)	0.0056 (4)	0.0076 (4)	0.0032 (5)
C15	0.0287 (5)	0.0277 (6)	0.0300 (5)	0.0044 (4)	0.0076 (4)	−0.0005 (4)
C16	0.0259 (5)	0.0254 (5)	0.0313 (6)	0.0057 (4)	0.0063 (4)	0.0019 (4)
C21	0.0293 (6)	0.0244 (5)	0.0353 (6)	0.0026 (4)	0.0099 (5)	0.0021 (5)
C22	0.0324 (6)	0.0314 (6)	0.0383 (6)	−0.0004 (5)	0.0078 (5)	0.0018 (5)
C23	0.0304 (6)	0.0328 (7)	0.0544 (8)	−0.0012 (5)	0.0123 (6)	0.0049 (6)
C24	0.0419 (7)	0.0304 (6)	0.0522 (8)	0.0017 (5)	0.0264 (6)	0.0075 (6)
C25	0.0490 (8)	0.0402 (7)	0.0370 (7)	−0.0005 (6)	0.0166 (6)	0.0052 (6)
C26	0.0344 (6)	0.0365 (7)	0.0367 (7)	0.0003 (5)	0.0082 (5)	0.0047 (5)
C31	0.0278 (5)	0.0292 (6)	0.0270 (5)	0.0034 (4)	0.0061 (4)	0.0025 (4)
C32	0.0338 (6)	0.0314 (6)	0.0354 (6)	0.0047 (5)	0.0093 (5)	0.0008 (5)
C33	0.0464 (8)	0.0290 (6)	0.0400 (7)	0.0031 (5)	0.0074 (6)	−0.0016 (5)
C34	0.0422 (7)	0.0347 (7)	0.0389 (7)	−0.0082 (6)	0.0026 (5)	0.0026 (5)
C35	0.0297 (6)	0.0443 (8)	0.0402 (7)	−0.0026 (5)	0.0057 (5)	0.0018 (6)
C36	0.0287 (6)	0.0341 (6)	0.0364 (6)	0.0039 (5)	0.0071 (5)	−0.0005 (5)
C41	0.0314 (6)	0.0382 (7)	0.0322 (6)	0.0051 (5)	0.0100 (5)	−0.0004 (5)
C42	0.0365 (7)	0.0412 (7)	0.0420 (7)	0.0062 (6)	0.0164 (5)	0.0044 (6)
C43	0.0423 (7)	0.0414 (7)	0.0405 (7)	0.0105 (6)	0.0124 (6)	0.0039 (6)
C44	0.0307 (6)	0.0505 (8)	0.0324 (6)	0.0105 (6)	0.0055 (5)	−0.0059 (6)
C45	0.0319 (6)	0.0518 (9)	0.0433 (7)	−0.0012 (6)	0.0142 (5)	−0.0064 (6)
C46	0.0383 (7)	0.0389 (7)	0.0440 (7)	0.0006 (6)	0.0146 (6)	−0.0018 (6)

### Geometric parameters (Å, °)

**Table:**

F1—C24	1.3630 (15)	C22—C23	1.3870 (18)
F2—C34	1.3586 (16)	C22—H22	0.9500
O1—C1	1.2145 (16)	C23—C24	1.368 (2)
O2—C12	1.3673 (15)	C23—H23	0.9500
O2—C4	1.4230 (18)	C24—C25	1.368 (2)
O3—N1	1.221 (2)	C25—C26	1.3889 (18)
O4—N1	1.2209 (19)	C25—H25	0.9500
N1—C44	1.4698 (18)	C26—H26	0.9500
C1—C2	1.4764 (17)	C31—C32	1.3949 (17)
C1—C11	1.5063 (16)	C31—C36	1.3985 (17)
C2—C3	1.3243 (18)	C32—C33	1.3848 (19)
C2—H2	0.9500	C32—H32	0.9500
C3—C41	1.4630 (17)	C33—C34	1.377 (2)
C3—H3	0.9500	C33—H33	0.9500
C4—H4A	0.9800	C34—C35	1.372 (2)
C4—H4B	0.9800	C35—C36	1.3832 (19)
C4—H4C	0.9800	C35—H35	0.9500
C11—C12	1.3965 (18)	C36—H36	0.9500
C11—C16	1.4022 (17)	C41—C46	1.3939 (19)
C12—C13	1.3890 (17)	C41—C42	1.397 (2)
C13—C14	1.3939 (17)	C42—C43	1.3802 (19)
C13—H13	0.9500	C42—H42	0.9500
C14—C15	1.3930 (17)	C43—C44	1.384 (2)
C14—C21	1.4844 (16)	C43—H43	0.9500
C15—C16	1.3997 (16)	C44—C45	1.374 (2)
C15—H15	0.9500	C45—C46	1.381 (2)
C16—C31	1.4877 (17)	C45—H45	0.9500
C21—C26	1.3905 (18)	C46—H46	0.9500
C21—C22	1.3948 (18)		
			
C12—O2—C4	117.90 (11)	F1—C24—C23	118.25 (14)
O4—N1—O3	123.89 (14)	F1—C24—C25	118.59 (14)
O4—N1—C44	117.61 (15)	C23—C24—C25	123.16 (12)
O3—N1—C44	118.49 (14)	C24—C25—C26	118.00 (13)
O1—C1—C2	122.49 (12)	C24—C25—H25	121.0
O1—C1—C11	120.47 (11)	C26—C25—H25	121.0
C2—C1—C11	117.04 (11)	C25—C26—C21	121.16 (13)
C3—C2—C1	121.13 (13)	C25—C26—H26	119.4
C3—C2—H2	119.4	C21—C26—H26	119.4
C1—C2—H2	119.4	C32—C31—C36	118.13 (12)
C2—C3—C41	126.04 (13)	C32—C31—C16	119.80 (11)
C2—C3—H3	117.0	C36—C31—C16	122.07 (11)
C41—C3—H3	117.0	C33—C32—C31	121.69 (12)
O2—C4—H4A	109.5	C33—C32—H32	119.2
O2—C4—H4B	109.5	C31—C32—H32	119.2
H4A—C4—H4B	109.5	C34—C33—C32	117.75 (13)
O2—C4—H4C	109.5	C34—C33—H33	121.1
H4A—C4—H4C	109.5	C32—C33—H33	121.1
H4B—C4—H4C	109.5	F2—C34—C35	118.84 (13)
C12—C11—C16	119.70 (11)	F2—C34—C33	118.30 (13)
C12—C11—C1	117.89 (11)	C35—C34—C33	122.85 (13)
C16—C11—C1	122.35 (11)	C34—C35—C36	118.63 (12)
O2—C12—C13	123.57 (12)	C34—C35—H35	120.7
O2—C12—C11	115.19 (11)	C36—C35—H35	120.7
C13—C12—C11	121.16 (11)	C35—C36—C31	120.91 (12)
C12—C13—C14	119.49 (12)	C35—C36—H36	119.5
C12—C13—H13	120.3	C31—C36—H36	119.5
C14—C13—H13	120.3	C46—C41—C42	118.89 (12)
C15—C14—C13	119.48 (11)	C46—C41—C3	119.20 (13)
C15—C14—C21	119.62 (11)	C42—C41—C3	121.91 (12)
C13—C14—C21	120.87 (11)	C43—C42—C41	120.79 (13)
C14—C15—C16	121.49 (11)	C43—C42—H42	119.6
C14—C15—H15	119.3	C41—C42—H42	119.6
C16—C15—H15	119.3	C42—C43—C44	118.22 (14)
C15—C16—C11	118.55 (11)	C42—C43—H43	120.9
C15—C16—C31	118.84 (11)	C44—C43—H43	120.9
C11—C16—C31	122.61 (11)	C45—C44—C43	122.82 (13)
C26—C21—C22	118.52 (12)	C45—C44—N1	119.22 (13)
C26—C21—C14	120.55 (11)	C43—C44—N1	117.96 (14)
C22—C21—C14	120.83 (11)	C44—C45—C46	118.19 (13)
C23—C22—C21	120.84 (13)	C44—C45—H45	120.9
C23—C22—H22	119.6	C46—C45—H45	120.9
C21—C22—H22	119.6	C45—C46—C41	121.07 (14)
C24—C23—C22	118.31 (13)	C45—C46—H46	119.5
C24—C23—H23	120.8	C41—C46—H46	119.5
C22—C23—H23	120.8		
			
O1—C1—C2—C3	−10.8 (2)	C23—C24—C25—C26	−1.4 (2)
C11—C1—C2—C3	168.41 (13)	C24—C25—C26—C21	0.0 (2)
C1—C2—C3—C41	−176.23 (13)	C22—C21—C26—C25	1.2 (2)
O1—C1—C11—C12	−114.27 (15)	C14—C21—C26—C25	−175.06 (12)
C2—C1—C11—C12	66.55 (15)	C15—C16—C31—C32	41.00 (16)
O1—C1—C11—C16	62.98 (18)	C11—C16—C31—C32	−138.88 (12)
C2—C1—C11—C16	−116.20 (13)	C15—C16—C31—C36	−138.17 (12)
C4—O2—C12—C13	20.49 (19)	C11—C16—C31—C36	41.95 (17)
C4—O2—C12—C11	−162.74 (12)	C36—C31—C32—C33	−1.30 (19)
C16—C11—C12—O2	179.64 (11)	C16—C31—C32—C33	179.50 (12)
C1—C11—C12—O2	−3.03 (17)	C31—C32—C33—C34	−0.5 (2)
C16—C11—C12—C13	−3.51 (18)	C32—C33—C34—F2	−179.18 (12)
C1—C11—C12—C13	173.82 (11)	C32—C33—C34—C35	1.6 (2)
O2—C12—C13—C14	178.94 (12)	F2—C34—C35—C36	−179.97 (12)
C11—C12—C13—C14	2.35 (19)	C33—C34—C35—C36	−0.7 (2)
C12—C13—C14—C15	1.00 (18)	C34—C35—C36—C31	−1.2 (2)
C12—C13—C14—C21	−176.80 (11)	C32—C31—C36—C35	2.18 (19)
C13—C14—C15—C16	−3.23 (18)	C16—C31—C36—C35	−178.64 (12)
C21—C14—C15—C16	174.59 (11)	C2—C3—C41—C46	165.67 (14)
C14—C15—C16—C11	2.07 (17)	C2—C3—C41—C42	−14.6 (2)
C14—C15—C16—C31	−177.81 (11)	C46—C41—C42—C43	0.2 (2)
C12—C11—C16—C15	1.28 (17)	C3—C41—C42—C43	−179.47 (13)
C1—C11—C16—C15	−175.93 (11)	C41—C42—C43—C44	−1.1 (2)
C12—C11—C16—C31	−178.84 (11)	C42—C43—C44—C45	0.5 (2)
C1—C11—C16—C31	3.96 (18)	C42—C43—C44—N1	−179.77 (13)
C15—C14—C21—C26	41.97 (17)	O4—N1—C44—C45	−12.8 (2)
C13—C14—C21—C26	−140.23 (13)	O3—N1—C44—C45	166.21 (15)
C15—C14—C21—C22	−134.25 (13)	O4—N1—C44—C43	167.52 (15)
C13—C14—C21—C22	43.55 (17)	O3—N1—C44—C43	−13.5 (2)
C26—C21—C22—C23	−1.07 (19)	C43—C44—C45—C46	1.0 (2)
C14—C21—C22—C23	175.23 (12)	N1—C44—C45—C46	−178.73 (13)
C21—C22—C23—C24	−0.3 (2)	C44—C45—C46—C41	−1.9 (2)
C22—C23—C24—F1	−177.96 (12)	C42—C41—C46—C45	1.3 (2)
C22—C23—C24—C25	1.6 (2)	C3—C41—C46—C45	−178.97 (13)
F1—C24—C25—C26	178.13 (13)		

### Hydrogen-bond geometry (Å, °)

**Table:**

Cg1 is the centroid of the C11–C16 ring.

**Table:**

*D*—H···*A*	*D*—H	H···*A*	*D*···*A*	*D*—H···*A*
C25—H25···F1^i^	0.95	2.54	3.2165 (17)	129
C33—H33···Cg1^ii^	0.95	2.91	3.4748 (15)	119
C24—F1···Cg1^iii^	1.3630 (15)	3.9512 (12)	4.8373 (15)	123.40 (8)

Symmetry codes: (i) −*x*+1, −*y*+2, −*z*; (ii) *x*, *y*−1, *z*; (iii) −*x*+1, *y*+1/2, −*z*+1/2.
